# Supplementary material for: Human Cripto-1 and Cripto-3 Protein Expression in Normal and Malignant Settings That Conflicts with Established Conventions
Source: Cancers (Basel). 2024 Oct 23;16(21):3577. doi: 10.3390/cancers16213577 (PMC11545644; doi:10.3390/cancers16213577)
Supplement: Supplementary file 1 [file cancers-16-03577-s001.zip › Supplemental Figure Legends.pdf]

Supplementary Figure Legends

Supplemental Figure S1. Representative example of resulting titration curves for NCI in-house versus commercially available antibody binding to solid phased recombinant human CR1 or CR3. All antibody test reagents initially diluted to [0.1mg/ml] before serially diluted in HFBTS (1:100 – 1:204,800). **A:** Solid phased R&D System recombinant human CR1 [50ng/50µl/well]. **B**: Solid phased MyBioSource recombinant human CR3 [50ng/50µl/well]. Decoding test antibodies: Blue – anti-CR1 NCI 5G1-1 MoAb, Red – anti-CR3 NCI G11-2 MoAb, Gray – anti-CR1/CR3 (Pan Rx) NCI 17G2-1 MoAb, Yellow – Santa Cruz anti-CR1 (Cat# sc376448), Light Blue – R&D Systems anti-CR1 (Cat# MAB2772), Green – CUSABIO anti-CR3 (Cat# CSB-PA302689), Black – Abcam anti-CR1 (Cat# ab10391), and Fushia – Abcam anti-CR1 (Cat# ab133236).

Supplemental Figure S2. IHC detection of CR1/CR3 in human tumor tissue and ranking of Grade, TMN_M, TMN_N, and TMN_T values based on staining intensity given almost significant/significant deltas. **A:** Discriminate ki67 grade levels in breast cancer tissue base on CR1 vessel expression, $p = 0.07$. **B:** TMN_M value in colon CA tissue inversely relates to CR3 tumor staining intensity, $p = 0.055$. **C:** TMN_N values in colon CA tissue inversely relates to CR3 tumor staining intensity, $p = 0.058$. **D:** TMN_T values in prostate CA tissue inversely related to CR3 tumor staining intensity, $p = 0.013$ (significant).

Supplemental Figure S3. Native uncropped WB gels assess CR1 vs CR3 expression in human tumor cell lines and an immortalized endothelial cell. Lane 1 - MW Std, Lane 2 - MCF7, Lane 3 - MDA-MB231, Lane 4 - HepG2, Lane 5 - HT29, Lane 6 - A549, and Lane 7 - hmVEC.
