# Supplementary material for: Human Cripto-1 and Cripto-3 Protein Expression in Normal and Malignant Settings That Conflicts with Established Conventions
Source: Cancers (Basel). 2024 Oct 23;16(21):3577. doi: 10.3390/cancers16213577 (PMC11545644; doi:10.3390/cancers16213577)
Supplement: Supplementary file 1 [file cancers-16-03577-s001.zip › Supplemental Figure S1.pptx]

## Slide 1
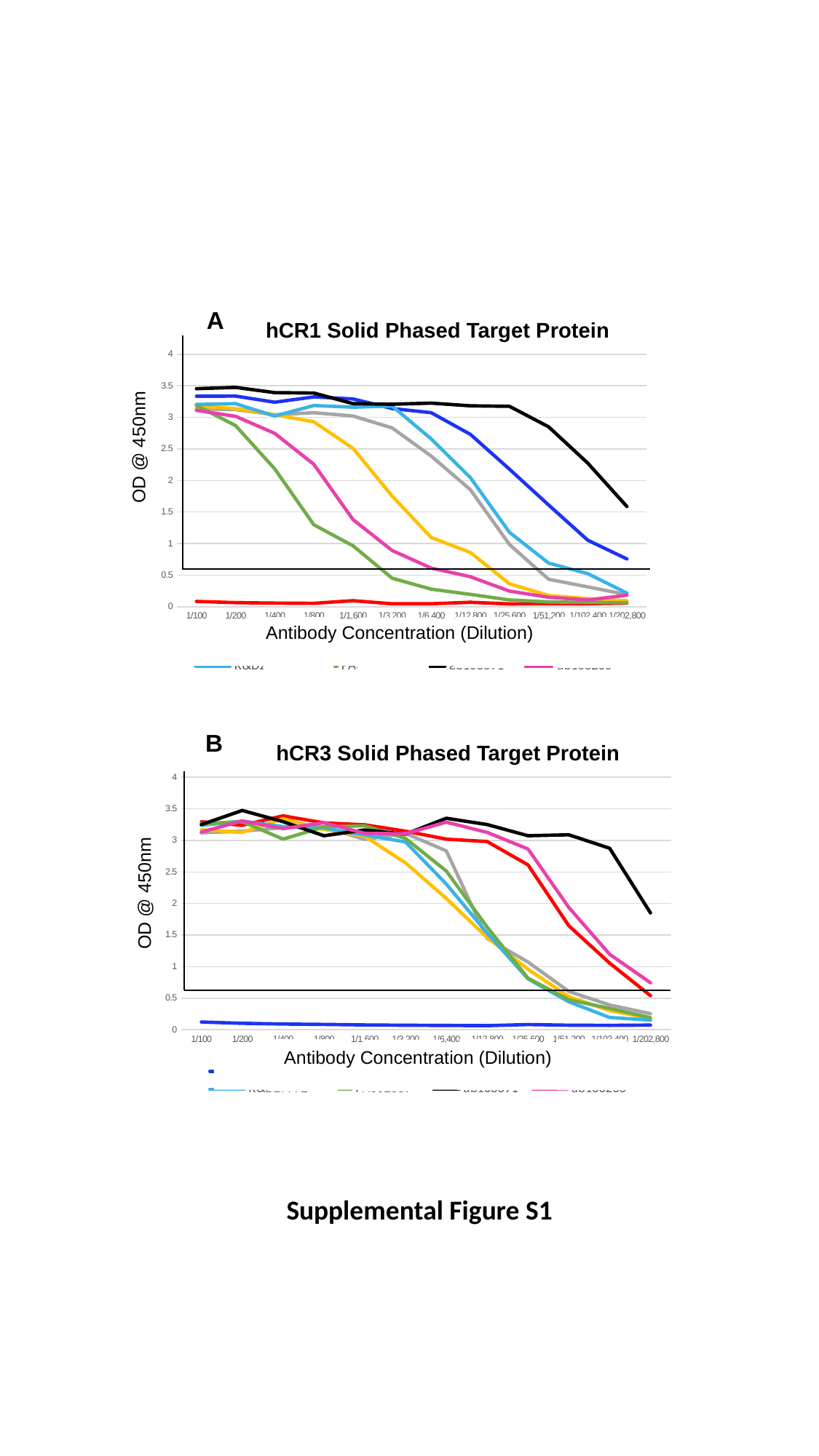

### Chart: Antibody Titration On Solid Phased R&D Systems Recombinant Human Cripto-1 [50ng/50ul/well]
| Category | 5G1-1 | GS 5G11-2 | GS 17G2-1 | SC376448 | R&D27772 | PA302689 | ab108391 | ab133236 |
|---|---|---|---|---|---|---|---|---|
| 1/100 | 3.335 | 0.085 | 3.134 | 3.179 | 3.205 | 3.188 | 3.454 | 3.11 |
| 1/200 | 3.337 | 0.066 | 3.124 | 3.139 | 3.217 | 2.87 | 3.475 | 3.018 |
| 1/400 | 3.24 | 0.058 | 3.041 | 3.043 | 3.021 | 2.185 | 3.392 | 2.747 |
| 1/800 | 3.324 | 0.055 | 3.074 | 2.929 | 3.188 | 1.298 | 3.385 | 2.257 |
| 1/1,600 | 3.292 | 0.096 | 3.022 | 2.51 | 3.161 | 0.966 | 3.218 | 1.382 |
| 1/3.200 | 3.14 | 0.047 | 2.833 | 1.753 | 3.179 | 0.452 | 3.208 | 0.891 |
| 1/6,400 | 3.074 | 0.048 | 2.387 | 1.097 | 2.659 | 0.279 | 3.226 | 0.613 |
| 1/12,800 | 2.731 | 0.071 | 1.854 | 0.86 | 2.046 | 0.196 | 3.183 | 0.476 |
| 1/25,600 | 2.179 | 0.045 | 0.985 | 0.363 | 1.178 | 0.108 | 3.175 | 0.248 |
| 1/51,200 | 1.612 | 0.048 | 0.435 | 0.178 | 0.692 | 0.077 | 2.85 | 0.151 |
| 1/102,400 | 1.054 | 0.048 | 0.315 | 0.128 | 0.524 | 0.068 | 2.275 | 0.108 |
| 1/202,800 | 0.757 | 0.058 | 0.192 | 0.095 | 0.217 | 0.066 | 1.587 | 0.183 |A
hCR1 Solid Phased Target Protein
OD @ 450nm
Antibody Concentration (Dilution)
NCI 17G2-1
SC376448
NCI 5G1-1
NCI 5G11-2
ab108391
ab133236
PA302689
MAB2772
### Chart: Antibody Titration On Solid Phased MyBioSource Recombinant Human Cripto-3 [50ng/50ul/well]
| Category | 5G1-1 | GS 5G11-2 | GS 17G2-1 | SC376448 | R&D27772 | PA302689 | ab108391 | ab133236 |
|---|---|---|---|---|---|---|---|---|
| 1/100 | 0.122 | 3.294 | 3.12 | 3.167 | 3.238 | 3.264 | 3.249 | 3.131 |
| 1/200 | 0.102 | 3.238 | 3.145 | 3.125 | 3.304 | 3.296 | 3.473 | 3.307 |
| 1/400 | 0.09 | 3.388 | 3.201 | 3.343 | 3.218 | 3.019 | 3.296 | 3.186 |
| 1/800 | 0.084 | 3.276 | 3.214 | 3.174 | 3.194 | 3.213 | 3.073 | 3.278 |
| 1/1,600 | 0.076 | 3.246 | 3.015 | 3.064 | 3.085 | 3.23 | 3.164 | 3.111 |
| 1/3.200 | 0.071 | 3.145 | 3.11 | 2.643 | 2.974 | 3.03 | 3.098 | 3.105 |
| 1/6,400 | 0.068 | 3.019 | 2.834 | 2.079 | 2.308 | 2.514 | 3.35 | 3.288 |
| 1/12,800 | 0.064 | 2.981 | 1.445 | 1.461 | 1.532 | 1.626 | 3.25 | 3.126 |
| 1/25,600 | 0.082 | 2.612 | 1.072 | 0.957 | 0.806 | 0.816 | 3.073 | 2.863 |
| 1/51,200 | 0.072 | 1.647 | 0.607 | 0.524 | 0.443 | 0.475 | 3.088 | 1.937 |
| 1/102,400 | 0.069 | 1.055 | 0.392 | 0.303 | 0.194 | 0.338 | 2.875 | 1.195 |
| 1/202,800 | 0.074 | 0.539 | 0.254 | 0.182 | 0.152 | 0.19 | 1.85 | 0.744 |B
hCR3 Solid Phased Target Protein
OD @ 450nm
NCI 17G2-1
SC376448
NCI 5G1-1
NCI 5G11-2
Antibody Concentration (Dilution)
ab108391
ab133236
PA302689
MAB2772
Supplemental Figure S1
