# Supplementary material for: Human Cripto-1 and Cripto-3 Protein Expression in Normal and Malignant Settings That Conflicts with Established Conventions
Source: Cancers (Basel). 2024 Oct 23;16(21):3577. doi: 10.3390/cancers16213577 (PMC11545644; doi:10.3390/cancers16213577)
Supplement: Supplementary file 1 [file cancers-16-03577-s001.zip › Supplemental Figure S2.pptx]

## Slide 1
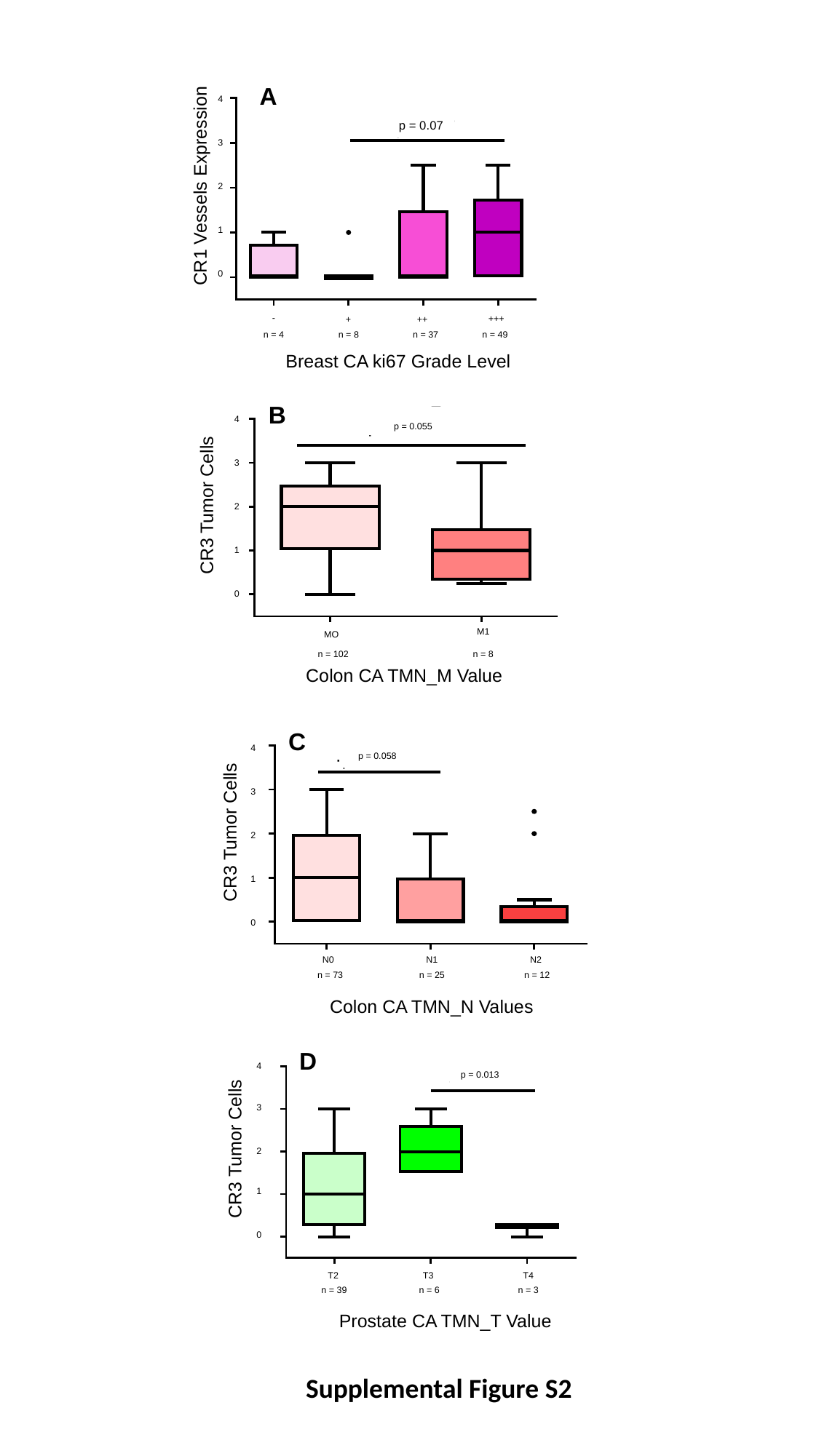

Breast CA ki67 Grade Level
A
4
3
2
1
0
p = 0.07
 CR1 Vessels Expression
-
+++
++
+
n = 4
n = 8
n = 37
n = 49
Breast CA ki67 Grade Level
 Colon CA TMN_M Value
B
4
3
2
1
0
p = 0.055
 CR3 Tumor Cells
M1
MO
n = 102
n = 8
Colon CA TMN_M Value
 Colon CA TMN_N Value
C
p = 0.058
4
3
2
1
0
p = 0.058
 CR3 Tumor Cells
N0
N1
N2
n = 73
n = 25
n = 12
Colon CA TMN_N Values
D
4
3
2
1
0
p = 0.013
CR3 Tumor Cells
T2
T3
T4
n = 39
n = 6
n = 3
 Prostate CA TMN_T Value
Supplemental Figure S2
