# Supplementary figures and images for: Human Cripto-1 and Cripto-3 Protein Expression in Normal and Malignant Settings That Conflicts with Established Conventions
Source: Cancers (Basel). 2024 Oct 23;16(21):3577. doi: 10.3390/cancers16213577 (PMC11545644; doi:10.3390/cancers16213577)

## Slide 1
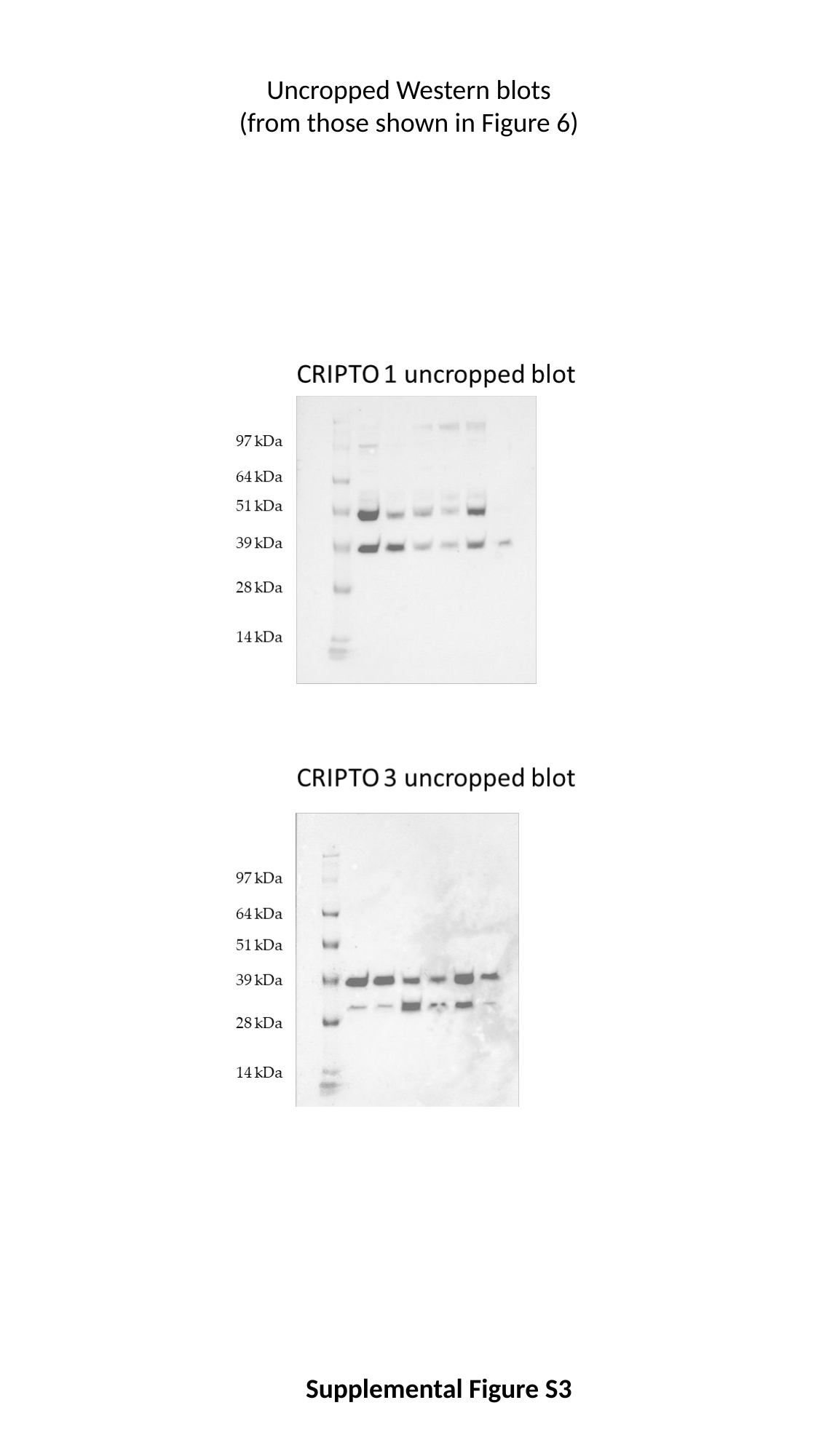

Uncropped Western blots
(from those shown in Figure 6)
Supplemental Figure S3

Supplement: Supplementary file 1 [file cancers-16-03577-s001.zip › Supplemental Figure S3.pptx]
